# A Pneumatic Soft Glove System Based on Bidirectional Bending Functionality for Rehabilitation

**DOI:** 10.3390/biomimetics10030129

**Published:** 2025-02-21

**Authors:** Xiaohui Wang, Qinkun Cheng, Zhifeng Wang, Yongxu Lu, Zhaowei Zhang, Xingang Zhao

**Affiliations:** 1 State Key Laboratory of Robotics, Shenyang Institute of Automation, Chinese Academy of Sciences, Shenyang 110016, China; xiaohuiw@mail.ustc.edu.cn (X.W.); chengqinkun@mail.ustc.edu.cn (Q.C.); wangzhifeng@sia.cn (Z.W.); luyongxu@sia.cn (Y.L.); 2 University of Chinese Academy of Sciences, Beijing 100049, China

**Keywords:** pneumatic soft glove, bidirectional bending actuator, rehabilitation gestures, assisted grip

## Abstract

Stroke-related hand dysfunction significantly limits the ability to perform daily activities. Pneumatic soft gloves can provide rehabilitation training and support for individuals with impaired hand function, enhancing their independence. This paper presents a novel pneumatic soft robotic system for hand rehabilitation featuring bidirectional bending actuators. The system comprises a pneumatic soft glove and a pneumatic control platform, enabling various rehabilitation gestures and assisting with finger grasping. The main bending module of the pneumatic soft actuator features a three-stage cavity structure, allowing for a wider range of finger rehabilitation training gestures and greater bending angles. The reverse-bending module uses a trapezoidal cavity design to enhance the reverse-bending capability, effectively facilitating finger extension motion. The pneumatic control platform is simple to set up, but effectively controls the actuators of the soft glove, which enables both main and reverse bending. This allows individuals with hand impairments to perform various gestures and grasp different objects. Experiments demonstrate that the pneumatic soft glove has a measurable load capacity. Additionally, the pneumatic soft glove system is capable of executing single-finger movements, a variety of rehabilitation gestures, and the ability to grasp different objects. This functionality is highly beneficial for the rehabilitation of individuals with hand impairments.

## 1. Introduction

With the increasing global aging population, stroke has become the third-leading cause of death and disability worldwide [1]. Approximately 55% to 75% of stroke survivors experience persistent motor impairments in the upper limb and hand [2,3]. Stroke patients often struggle to control the affected areas of their body, significantly impacting their quality of life, ability to perform activities of daily living (ADLs), and even their psychological well-being, ultimately reducing their independence [4,5]. Among the various body parts involved, the hand plays a crucial role in physical interactions between humans and their environment [6,7], which has led to an increasing demand for hand rehabilitation among patients. However, traditional manual rehabilitation methods are costly, inefficient, and vary widely in quality, with a severe shortage of therapists [8,9]. As a result, robot-assisted rehabilitation has garnered increasing attention as a promising approach for supporting hand function recovery in stroke patients [10]. Studies have shown that robot-assisted repetitive task training can effectively aid in the recovery of hand function [11,12,13].

Traditional hand rehabilitation robots typically use rigid structures. These rigid robots were among the first to be developed for rehabilitation and assistive applications, as their rigid mechanical drive systems can transmit high forces with high precision [14]. Rigid robots for hand rehabilitation primarily use motors to drive finger joints. The average weight of these devices is approximately 500 g. They are relatively large compared with the size of the human hand, making them uncomfortable to wear and difficult to carry. Additionally, they pose safety risks, potentially causing further injury to patients [15,16]. Rigid structures reduce the biomimetic quality of robots, limiting their therapeutic potential. To overcome these limitations, soft actuators made from hyperelastic materials have emerged as novel types of continuous biomimetic robots applied in finger rehabilitation and assistive movements [17]. Among these, pneumatic soft hand rehabilitation robots have become a key focus of research because of their light weight, safety, flexibility, portability, and excellent biocompatibility [18,19], making them highly suitable for hand rehabilitation and assistance [20].

Polygerinos et al. introduced a pneumatic network actuator made from hyperelastic materials featuring an integrated channel design that incorporates a soft actuator into an open glove made of flexible material. Their study demonstrated the actuator’s ease of use and feasibility of fist formation [21]. However, this type of soft actuator can only produce curved contours resembling arcs, limiting its ability to replicate the complex curvature of finger flexion. Connolly et al. designed a fiber-reinforced soft actuator that mimics biological joints by extending, expanding, twisting, and pressurizing sections made of different cylindrical elastic tubes [22]. Although the actuator’s bending appears to simulate the flexion of three joints, it is actually controlled by a single actuation, meaning all joints must bend simultaneously. Feng et al. introduced a pneumatic soft actuator with a three-stage cavity structure that can independently drive the three joints of a finger [23]. However, this actuator is limited to flexion movement and cannot facilitate the extension movement of the finger. Yap et al. introduced a stiffness-adjustable pneumatic soft actuator designed to mimic the natural flexion of the human hand under pneumatic pressure [24]. On the basis of the anatomical structure of the human finger, J. Wang et al. developed a segmented pneumatic soft actuator that conforms to the finger’s contours, and embedded an internal bladder into the cavity to improve airtightness [25]. However, both of these soft actuators are unable to support finger extension movement. F. Wang et al. introduced a bidirectional pneumatic soft rehabilitation glove that combines both rehabilitation and assistive functions. The main body of the soft actuator, responsible for both main and reverse bending, uses rectangular solids. The joints of the main bending module feature trapezoidal air cavities, while the reverse-bending module uses triangular air cavities at its joints. This glove mimics human finger joints and offers four distinct rehabilitation postures [26]. However, the design of the main bending module limits the range of both finger flexion and extension angles. Simultaneously, the design of the reverse-bending module affects the reverse-bending angle of the finger and results in a smaller fingertip force during reverse bending.

Soft actuators have attracted substantial interest; however, their fluid-driven systems have been relatively overlooked [27,28]. Pneumatic actuation in soft hand robots is typically powered by compressed air, with air pumps serving as the system’s air source, and various valves control the direction of airflow [29,30,31]. As soft hand rehabilitation robot systems become more complex, there is an increasing demand for precise control of actuator movements. In this context, relays and solenoid valves play crucial roles [32,33]. Several studies on soft hand rehabilitation robots have discussed the use of solenoid valve-based systems [34,35,36]. Skorina et al. designed a valve system where the inlet is directly connected to a compressed air pump, the outlet is linked to the soft actuator, and the exhaust port is open to the atmosphere [37]. T. Wang et al. employed a dual two-position, two-way solenoid valve (2/2-way solenoid valve) system to reduce energy consumption and increase the valve lifespan [38]. Young et al. used 26 switch valves and 5 proportional valves to control a pneumatic soft hand robot, enabling simultaneous control of multiple actuators [31]. Overall, there are few studies on control platforms specifically designed for managing the bidirectional bending of soft actuators.

This paper presents a bidirectional pneumatic soft glove system, comprising both a pneumatic soft glove and a pneumatic control platform. The main bending module of the bidirectional pneumatic soft glove features a three-stage cavity structure that mimics the finger joints. To allow reverse bending without interfering with the main bending movement, the reverse-bending module uses a trapezoidal cavity design. Additionally, a simple and easily replicable programmable pneumatic control platform was designed to manage the complex movements of the soft glove. First, an analysis of the soft actuator revealed that the pneumatic soft glove has a measurable load capacity. Then, through rehabilitation experiments in which the glove was worn on both a prosthetic hand model and a human hand, its ability to perform various rehabilitation gestures and assist patients in grasping different objects was demonstrated. This pneumatic soft glove system enables both finger flexion and extension, supporting individuals with hand impairments in achieving greater independence in ADLs.

## 2. Design and Fabrication of the Soft Finger Actuator

### 2.1. The Structure Design of the Actuator

**Figure 1:**
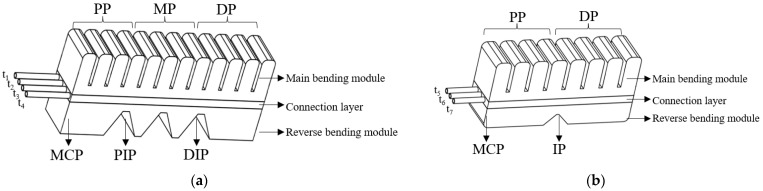
The structure of the soft actuators. (**a**) structure of the other fingers (except for the thumb); (**b**) structure of the thumb.

Owing to the complex interactions between tendons, ligaments, muscles, and bones, the human hand has at least 20 degrees of freedom [39], enabling it to perform intricate movements. The design of our pneumatic soft actuator is inspired by the soft actuator developed by Polygerinos et al. and J. Wang et al. [21,25]. To enable finger extension, a reverse-bending module was added. The design is inspired by J. Wang et al., drawing on their key features and principles. This adaptation makes our reverse-bending module better aligned with the human hand, improving the fit of the soft glove and the hand’s natural movement. Building on the design by Polygerinos et al., we have improved the structure and internal cross-sectional shape of the air cavity in the main bending module, as well as the spacing between the cavities, etc. These modifications enhance the bending angle of the main bending module and provide sufficient space for the reverse bending. Following the anatomical structure of the human hand, a pneumatic network with three joints was designed, making the actuator more closely resemble the human hand (excluding the thumb), as shown in Figure 1a. On the basis of the structure and length of the thumb, a pneumatic network with two joints was designed, as shown in Figure 1b.

Except for the thumb, the soft actuator for each finger consists of three phalanges—the distal phalanx (DP), middle phalanx (MP), and proximal phalanx (PP)—corresponding to the distal interphalangeal (DIP), proximal interphalangeal (PIP), and metacarpophalangeal (MCP) joints, respectively. The main bending module features a three-stage cavity structure, with each phalanx consisting of four air cavities, for a total of twelve cavities. Each air cavity features a chamfered cross-sectional design [23], with an axial wall thickness of 2 mm and a lateral wall thickness of 3 mm. This design ensures a maximum bending angle during axial flexion, meeting the requirements for the angle of finger flexion while also providing sufficient fingertip force. The soft actuator can perform single-joint movements or multi-joint coordinated motions. By adjusting the airflow through different combinations of the t_1_, t_2_, and t_3_ tubes, various rehabilitation gestures can be achieved. Without affecting the main bending module, the reverse-bending module, which is designed with a trapezoidal cross section, provides greater space for reverse bending, compared to the rectangular cross-sectional design by F. Wang et al. [26]. By inflating the t_4_ tube, the reverse-bending module generates adequate force for finger extension while preventing any secondary injury to the finger.

The thumb consists of the distal phalanx (DP) and proximal phalanx (PP), corresponding to the interphalangeal joint (IP) and metacarpophalangeal joint (MCP) of the thumb. The main bending module of the thumb contains eight air cavities distributed across two phalanges. The reverse-bending module is also designed with trapezoidal cross-sectional air cavities. By adjusting the airflow through different combinations of the t_5_ and t_6_ tubes, different joint flexions of the thumb can be achieved. By inflating the t_7_ tube, extension movement of the thumb can be achieved. Without compromising the degrees of freedom of the finger, the thumb plays a crucial role in grasping objects of various shapes.

Therefore, the designed pneumatic soft glove can perform a wider range of rehabilitation gestures and assist patients with hand impairments in grasping tasks during daily activities.

### 2.2. Structural Theoretical Analysis

Pneumatic soft actuators are made of silicone rubber and exhibit nonlinear characteristics. When appropriate pressure is applied, the actuator can extend to several times its original size. Once the pressure value drops to zero, it returns to its initial state. Abaqus simulations of the soft actuator were conducted via the Yeoh model for third-order hyperelastic materials to analyze its mathematical behavior and complete the fabrication of the soft actuator. The geometric parameters and values of the soft actuator are provided in Table A1.

#### 2.2.1. Bending Angle of the Main Bending Module

**Figure 2:**
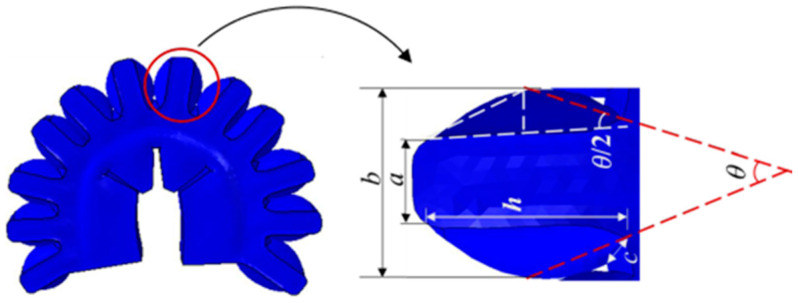
Parameters of a single air cavity of the main bending module after expansion.

The deformation of a single air cavity in the main bending module, along with its re-lated parameters, is shown in Figure 2. The initial length of a single air cavity is *a*, whereas its extended length after deformation is *b*. The height and width of the cavity are denoted *h* and *c*, respectively. The total bending angle after deformation is *θ*. There-fore, the deformation can be expressed in terms of known parameters such as *a*, *c* and *h*.

After pressurization, the deformed section of the air cavity can be approximated as an isosceles triangle. By drawing a perpendicular line within the triangle, a smaller triangle containing angle θ/2 is formed. For the smaller triangle, the lengths of the perpendicular sides are denoted h/2, (a−b)/2. According to the principle of equal areas in triangles, the following relationship is derived:

(1) $$\sin\frac{\theta }{2}=\frac{b-a}{\sqrt{{\left(b-a\right)}^{2}+h^{2}}}$$

From this, the following equation for θ can be derived:

(2) $$\theta =\arcsin(2\frac{(b-a)h}{{\left(b-a\right)}^{2}+h^{2}})+\varepsilon ,$$

The error due to factors such as the environment, material properties, and fabrication precision is represented by ε.

Since the final posture of the soft actuator results from the coupling between individual air cavities [23], the geometric relationship between the internal air pressure and the bending angle of each cavity can be inferred. It is assumed that the gas behaves as an ideal gas and that the ambient temperature remains constant throughout the experiment. The soft actuator deforms and bends upon inflation. According to Avogadro’s hypothesis, at constant temperature (T) and pressure, the volume of gas in a single cavity is directly proportional to its mass. Within the elastic deformation stage, the stress–strain relationship of Ecoflex 00-50 follows Hooke’s law, where Young’s modulus (*E*), shear modulus (*G*), and Poisson’s ratio (μ) remain constant [40,41]. Theoretically, the elastic constants *E*, *G*, and μ of an isotropic material have the following relationship:

(3) E=2G(1+μ)

The change in volume during the deformation process is referred to as the bulk modulus (*K*):

(4) $$K=\frac{\Delta P}{\Delta V/V_{0}}=\frac{Pahc}{\Delta V}$$

The relationship between the bulk modulus, tensile modulus, and Poisson’s ratio is ex- pressed as follows:

(5) E=3K(1−2μ)

where ΔP represents the variation in pressure, P is the pressure, ΔV represents the variation in gas volume, V represents the initial value of the gas volume, and the values of *E* and *G* are shown in Table A1.

When the cavity inflates, it can be approximated as a semi-ellipsoid, as shown in Figure 2. Therefore, the incremental volume of a single cavity can be expressed as:

(6) $$V=\frac{1}{3}\pi hc(b-a)$$

From Equations (3)–(6), we obtain:

(7) $$\frac{\pi hc(b-a)}{3}=Pahc\frac{9G-3E}{EG},$$

thus:

(8) $$b-a=\frac{3Pa(9G-3E)}{\pi EG}$$

Substituting Equation (8) into Equation (2) yields the relationship between the bending angle θ and the input air pressure P:

(9) $$\theta =\arcsin(\frac{2Pkh}{P^{2}k^{2}+h^{2}})+\varepsilon ,$$

where:

(10) $$k=\frac{3a(9G-3E)}{\pi EG}$$

#### 2.2.2. Bending Angle of the Reverse-Bending Module

**Figure 3:**
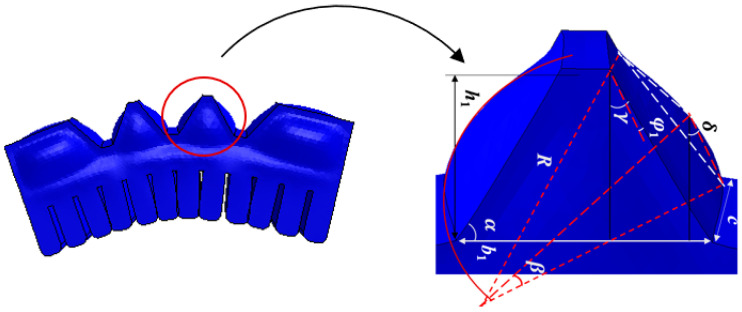
Parameters of a single air cavity of the reverse-bending module after expansion.

The central section of the reverse-bending module is designed with an air cavity structure in the shape of an isosceles trapezoid, with identical base angles, as shown in Figure 1a. The deformed state of a single trapezoidal air cavity after inflation and its related parameters are shown in Figure 3. The trapezoidal module has a short side of *a*_1_, base length of *b*_1_, height of *h*_1_, and width of *c*. The angle of the base of the trapezoid is α, and half of the bending angle after deformation is $\varphi_{1}$, where $\varphi_{1}$ is the difference between the angle γ between the original trapezoidal hypotenuse and the vertical direction, and the angle δ between the vertex of the expanded triangle on the arc and the vertical direction, i.e., $\varphi_{1}=\gamma -\delta$.

After pressurization, part of the air cavity approximates a circular arc. Let the radius of the resulting sector be *r*, the corresponding central angle be 2β, and the length of the trapezoid’s slanted side be *t*. The following relationship can be derived:

(11) $$t=\frac{h_{1}}{\sin\alpha }$$

(12) $$\beta =2\varphi_{1}$$

(13) $$r=\frac{t}{2\sin\beta }$$

After inflation, the chord volume on one side, denoted $V_{s}$, is given by:

(14) $$V_{s}=V_{S}-V_{T}=\beta r^{2}c-\frac{t}{2}cr\cos\beta$$

where $V_{S}$ represents the volume of the sector formed by the circular arc and $V_{T}$ denotes the volume of the triangular prism formed by the intersection of the sector and the trapezoid.

The volume of the original trapezoid is $V_{t}$:

(15) $$V_{t}=\frac{1}{2}(a_{1}+b_{1})h_{1}c$$

When the cavity inflates, the incremental volume of a single cavity, denoted $V^{\prime }$, can be expressed as:

(16) $$V^{\prime }=2V_{s}=2\beta r^{2}c-ctr\cos\beta$$

Similarly, on the basis of the relationship between the elastic parameters *E*, *G*, and the bulk modulus *K* during the material’s elastic deformation stage, the following expression is obtained:

(17) $$2(V_{S}-V_{T})=P^{\prime }V_{t}\frac{9G-3E}{EG}$$

Substituting Equations (15) and (16) into (17), we obtain:

(18) $$2\beta r^{2}-tr\cos\beta =\frac{P^{\prime }h_{1}(a_{1}+b_{1})(9G-3E)}{2EG}$$

Substituting Equations (11) and (13) into (18), we obtain:

(19) $$\frac{\beta -\cos\beta \sin\beta }{\sin^{2}\beta }=k^{\prime },$$

where:

(20) $$k^{\prime }=\frac{P^{\prime }(a_{1}+b_{1})(9G-3E)}{EGh_{1}}\sin^{2}\alpha$$

where $P^{\prime }$ is the pressure and the values of *E*, *G* and the other parameters are shown in Table A1.

When the inflation pressure is constant, $k^{\prime }$ can be treated as a constant, yielding the following expression:

(21) $$\frac{\beta -\cos\beta \sin\beta }{\sin^{2}\beta }=k^{\prime }$$

(22) $$\beta -\cos\beta \sin\beta =k^{\prime }\sin^{2}\beta$$

Taking the derivative of both sides of Equation (22) gives:

(23) $$1-(\cos^{2}\beta -\sin^{2}\beta )=2k^{\prime }\sin\beta \cos\beta$$

(24) $$\beta =\arctan k^{\prime }$$

Substituting Equation (12) into Equation (24), we obtain half of the bending angle after deformation, denoted $\varphi_{1}$:

(25) $$\varphi_{1}=\frac{\arctan k^{\prime }}{2}$$

Finally, we obtain the relationship between the bending angle φ of a single deformed trapezoidal gas chamber and the input air pressure $P^{\prime }$:

(26) $$\varphi =\arctan(\frac{P^{\prime }(a_{1}+b_{1})(9G-3E)}{EGh_{1}}\sin^{2}\alpha +\varepsilon$$

For the irregular trapezoidal sides of the reverse-bending module, the bending angle of a single irregular trapezoid after inflation is $\varphi_{1}$.

When the input air pressure is within the safe operating range of the soft actuator, this theoretical model can be used to calculate the initial input pressure. By adjusting the initial pressure, the desired finger bending angle can be achieved.

### 2.3. Fabrication of the Single-Finger Actuator

**Figure 4:**
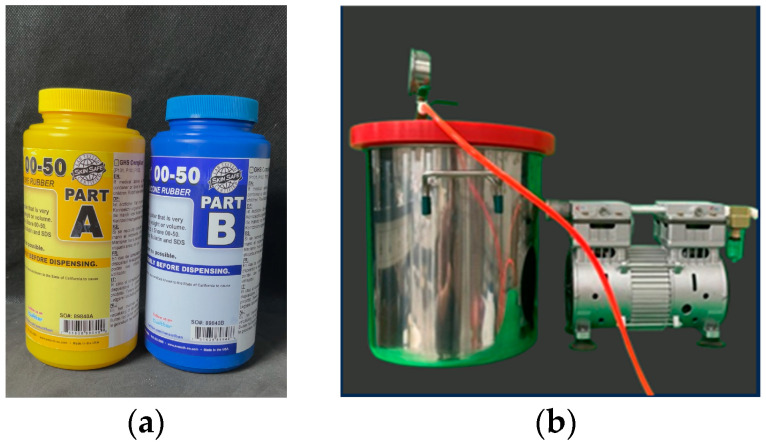
(**a**) Material of soft actuator-Ecoflex 00-50; (**b**) vacuum pump.

The soft actuator is made primarily of Ecoflex 00-50 silicone (Smooth-On Inc., Macungie, PA, USA), as shown in Figure 4a. The silicone was mixed at a 1A:1B weight ratio, thoroughly stirred, and then placed in a vacuum pump (Danhao oil-free vacuum pump) to eliminate air bubbles (Figure 4b) [42]. Afterward, the mixture was poured into a mold and left to cure for 3 h before demolding. After demolding, the layers were bonded together via a silicone adhesive (Smooth-On Silpoxy) and left to cure for one hour to ensure thorough bonding. To facilitate demolding, a release agent (Smooth-On Release 200) was evenly sprayed onto the inner surface of the mold 5 min before pouring. The material properties of Ecoflex 00-50 silicone are summarized in Table 1.

**Figure 5:**
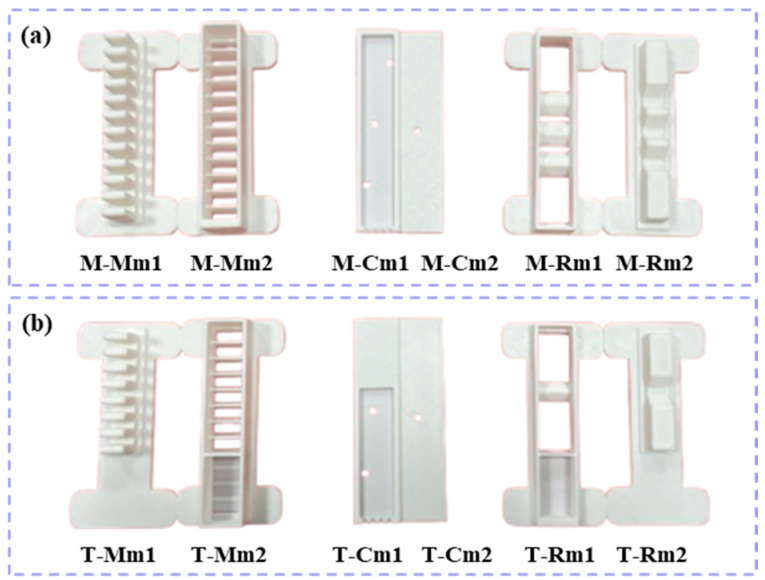
Finger molds. (**a**) Mold for index finger, middle finger, ring finger, and little finger; (**b**) mold for thumb.

The molds for the soft actuators were made from polylactic acid (PLA) and were printed with a Bambu Lab 3D printer, as shown in Figure 5. In this study, the middle finger is used as a representative example of the other fingers. Figure 5a,b display the molds for the middle finger and the thumb, respectively. In Figure 5a, the molds for the main bending module are labeled M-Mm1 and M-Mm2, the intermediate connecting layer module molds are labeled M-Cm1 and M-Cm2, and the reverse-bending module molds are labeled M-Rm1 and M-Rm2. In Figure 5b, the molds for the main bending module are labeled T-Mm1 and T-Mm2, the intermediate connecting layer module molds are labeled T-Cm1 and T-Cm2, and the reverse-bending module molds are labeled T-Rm1 and T-Rm2.

**Figure 6:**
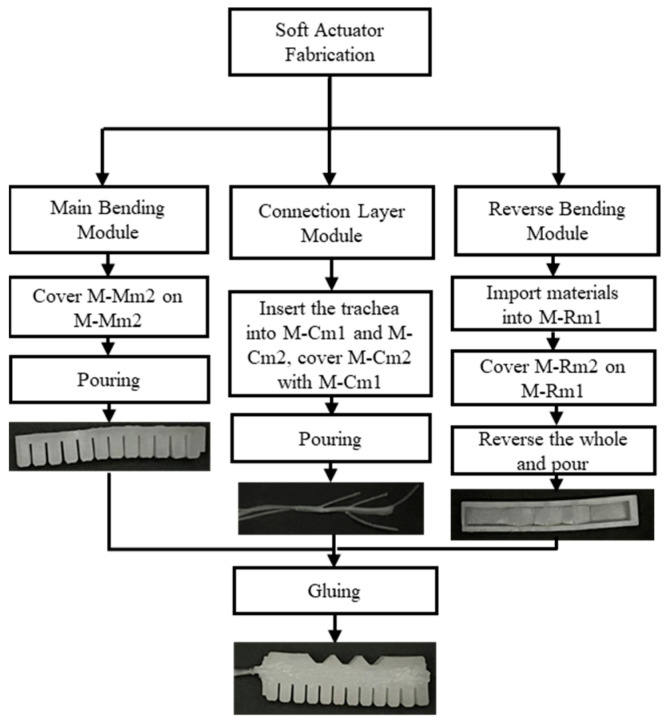
Fabrication process diagram of the middle finger of the soft actuator.

The fabrication process for the soft actuator of the middle finger is shown in Figure 6, which can be explained in the following three steps.

**Step I**: Make the main bending and reverse-bending modules. The main bending module is formed by combining molds M-Mm1 and M-Mm2, followed by casting. To prevent air bubbles or incomplete filling in the trapezoidal cavities during the casting of the reverse-bending module, we employed a two-step casting process. First, mold M-Rm1 is placed on a thin slice, and silicone material is poured into it. Then, mold M-Rm2 is securely positioned inside mold M-Rm1. Finally, the entire mold is inverted, and additional material is added to ensure full encapsulation.

**Step II**: Fabricating the intermediate layer and securing the soft tubing. The soft tube is inserted into the holes of molds M-Cm1 and M-Cm2, with one end extending approximately 15 cm beyond the lower edge of mold M-Cm1. Silicone material is then used to fill mold M-Cm1, after which mold M-Cm2 is placed over M-Cm1 and securely fixed.

**Step III**: After demolding, the main bending and reverse-bending sections are bonded together through the intermediate layer, completing the fabrication of the soft actuator.

The fabrication process for the thumb soft actuator follows the same steps as those described above.

## 3. Design of the Pneumatic Control Platform for Soft Glove

**Figure 7:**
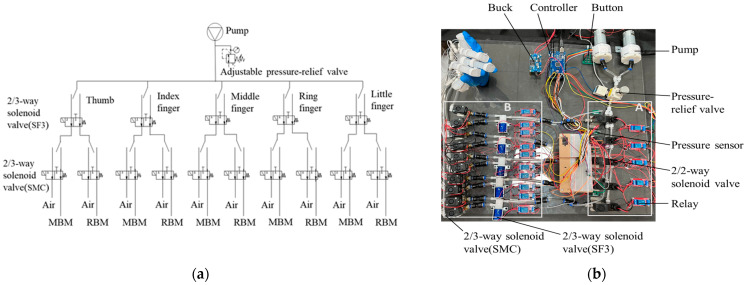
Design of the pneumatic system. (**a**) pneumatic circuit diagram; (**b**) pneumatic control platform.

Many soft robots have been developed to emulate the movement of human fingers [43], including pneumatic soft gloves. As a key component of soft robotics, the pneumatic system plays a critical role. In particular, pneumatic systems based on pumps and electromagnetic valves demonstrate excellent performance [44]. To achieve our design requirements with a simple and controllable pneumatic circuit, we developed a programmable pneumatic control platform, as illustrated in Figure 7b. This platform, which is based on a combination of pumps and electromagnetic valves, enables a soft robotic glove with bidirectional bending capability to assist patients with hand impairments in rehabilitation tasks. The model and origin of the components used in the pneumatic control platform are detailed in Table A2.

The working principle of the pneumatic system is illustrated in Figure 7a. The system consists of a main airway and a branch airway, as shown in Figure 7b(A,B).

The pneumatic control platform uses a micro air pump as the air source, with a maximum flow rate of 15 L/min and an output pressure range of −70 to 90 kPa. A pressure-relief valve is used as a mechanical safeguard to prevent excessive pressure and allows for adjustment of the input pressure. A DC-DC buck converter (input: 6.5–40 V, output: 5 V/3 A) provides +5 V to power the Arduino controller. The desired rehabilitation gesture or assistive function is selected via a 4 × 4 matrix keypad. The Arduino controller then sends signals to control the relays, which regulate the two-position, two-way solenoid valves (2/2-way solenoid valves), enabling the selection of the main airway for the corresponding finger. During this process, an air pressure sensor (0–100 kPa) measures the pressure in the airway, and the feedback data are used to adjust the inflation pressure through the pressure regulation module. Finally, the relays in the branch circuit control the two-position, three-way solenoid valves (SF3 and SMC) (2/3-way solenoid valves) to select either the main bending module (MBM) or reverse-bending module (RBM) of soft actuators, enabling human finger flexion or extension, thereby completing the rehabilitation exercises of the pneumatic soft glove. The system also realizes the maintaining of the rehabilitation gesture of the soft glove. During the hold state, the air pump and relays in the main airway are powered off, whereas the two-position, three-way solenoid valve (SMC) in the branch airway operates to maintain the gesture. After the hold state, the components in the branch circuit are de-energized. The 2/3 solenoid valves (SMC) are switched to vent to the atmosphere (Air), releasing the gas from the soft actuator.

Experiments demonstrated the practicality of the pneumatic control platform. Additionally, its versatility and scalability offer valuable insights for the drive and control of soft robots.

## 4. Experimental Verification

To validate the ability of the pneumatic soft glove in performing various rehabilitation gestures and assisting with grasping tasks, we conducted experiments with both a single soft actuator and a soft glove.

### 4.1. Characteristic Testing of the Single-Finger Actuator

#### 4.1.1. Bending Angle Measurement

To assess the impact of gravity on the bending angle and input pressure, tests were conducted in three orientations: horizontal, against the vertical direction of gravity (against gravity), and the direction of gravity (with gravity). As the soft actuator reaches its maximum bending angle without distortion, the air pressure value is continuously monitored and recorded in real-time via an air pressure sensor. In each direction, multiple experiments were conducted under each entity model, and the average values were taken. Kalman filtering was then applied for optimization to obtain the pressure values, which is the optimal air pressure value.

**Figure 8:**
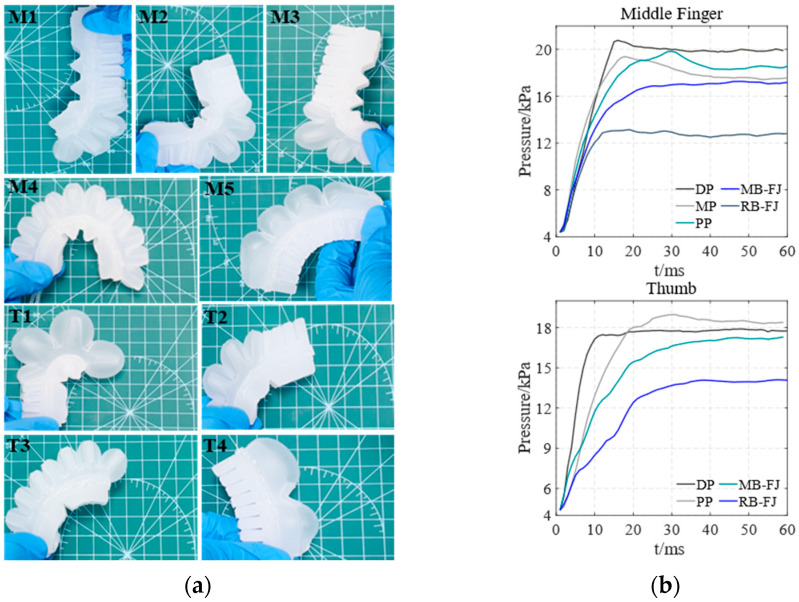
The main bending of the soft actuator’s finger joints and the overall reverse bending of the soft actuator in the horizontal direction. (**a**) entity models of the middle finger and thumb during bending; (**b**) air pressure values of the bending of the middle finger and thumb.

Figure 8 shows the entity models and air pressure values of both the main bending of the soft actuator’s finger joints and the overall reverse bending of the soft actuator in the horizontal direction. Figure 8a shows the entity models of the soft actuator in different bending states. M1–M5 represent the middle finger’s DP, MP, PP, and full joint in both main bending (MB-FJ) and reverse bending (RB-FJ), respectively. Similarly, T1–T4 represent the thumb’s DP, PP, and full joint in both main bending and reverse bending, respectively. The air pressure values corresponding to M1–M5 for the middle finger and T1–T4 for the thumb are shown in Figure 8b.

**Figure 9:**
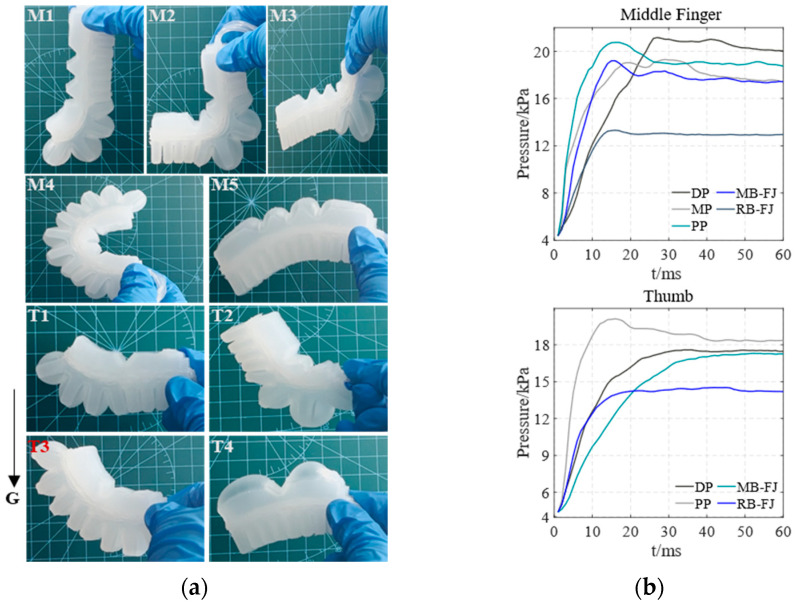
The main bending of the soft actuator’s finger joints and the overall reverse bending of the soft actuator in the direction of against gravity. (**a**) entity models of the middle finger and thumb during bending; (**b**) air pressure values of the bending of the middle finger and thumb.

**Figure 10:**
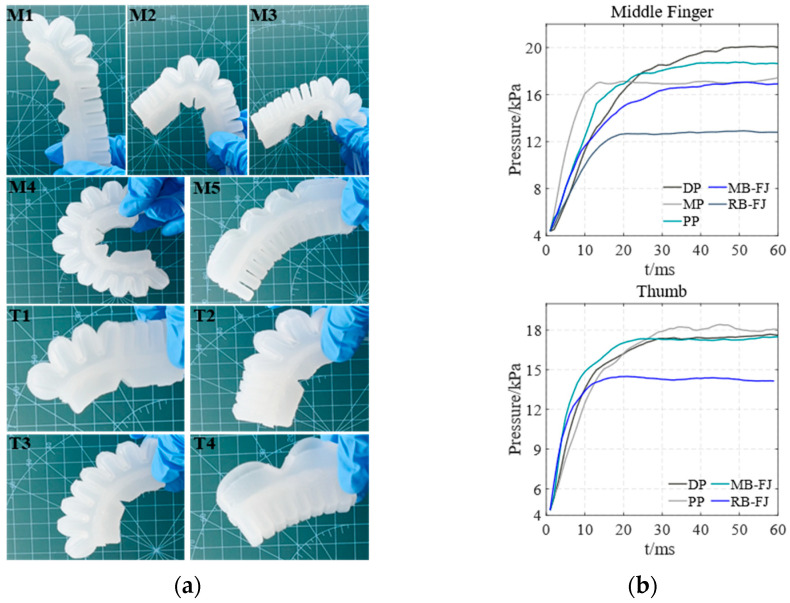
The main bending of the soft actuator’s finger joints and the overall reverse bending of the soft actuator in the direction of gravity: (**a**) entity models of the middle finger and thumb during bending; (**b**) air pressure values of the bending of the middle finger and thumb.

Figure 9 and Figure 10 illustrate the entity models and air pressure values of both the main bending of the soft actuator’s finger joints and the overall reverse bending of the soft actuator in both the direction of against gravity and the direction of gravity, respectively. The arrangement and correspondence of these images follow the same pattern as those in Figure 8.

By comparing Figure 8b, Figure 9b and Figure 10b, the optimal air pressure values for both the main bending of the soft actuator’s finger joints of the middle finger and thumb and the overall reverse bending of the soft actuator’s middle finger and thumb are obtained. The air pressure values at the maximum main and reverse-bending angles of the soft actuator are independent of its orientation. Therefore, the effect of gravity on the soft actuator can be considered negligible.

**Figure 11:**
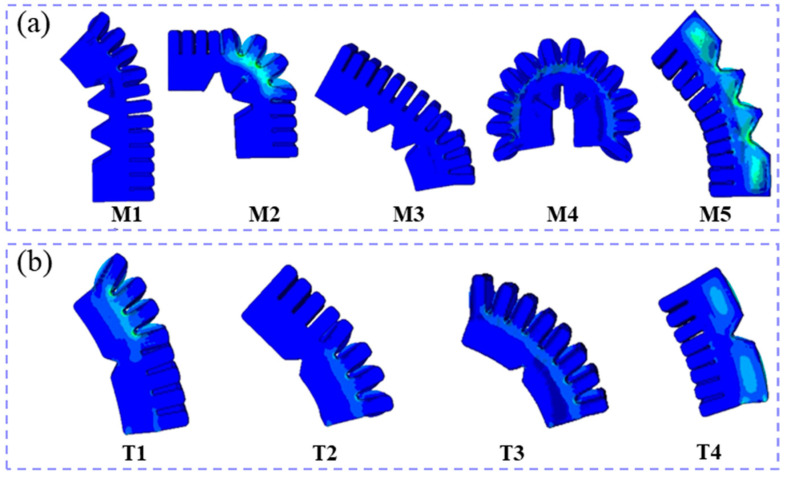
Abaqus simulation sequence diagrams of the soft actuator of the middle finger and the thumb under optimal air pressure. (**a**) middle-finger Abaqus simulation sequence diagrams; (**b**) thumb Abaqus simulation sequence diagrams.

At the optimal air pressure, we select the Yeoh model of a third-order hyperelastic material to describe the mathematical model of the soft actuator, as illustrated in Figure 11. The parameters of the Yeoh hyperelastic strain energy potential are as follows: C10 = 0.11, C20 = 0.02, and C30 = 6.07 × 10^−5^ [45]. The density of Ecoflex 00-50 is 1070 kg/m^3^.

**Figure 12:**
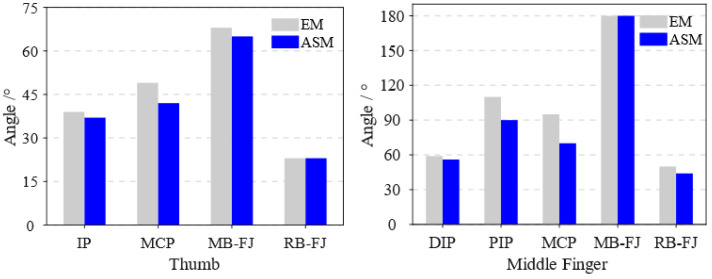
Bending angle values of the entity model and Abaqus simulation model of the soft actuators.

The bending angles of both the entity model (EM) shown in Figure 10a and the Abaqus simulation model (ASM) of the soft actuators are shown in Figure 12. The simulation models of the soft actuators, obtained through finite element analysis in Abaqus, closely match the entity models. For the thumb, the soft actuator can reach a maximum main bending angle of 68° and a reverse-bending angle of 23°. The middle finger can achieve a maximum main bending angle of 180° and a maximum reverse-bending angle of 50°. The main and reverse-bending angles of each joint in soft actuators meet the movement requirements for hand function in daily human activities [46]. Subsequently, all experiments were conducted in the direction aligned with gravity.

**Figure 13:**
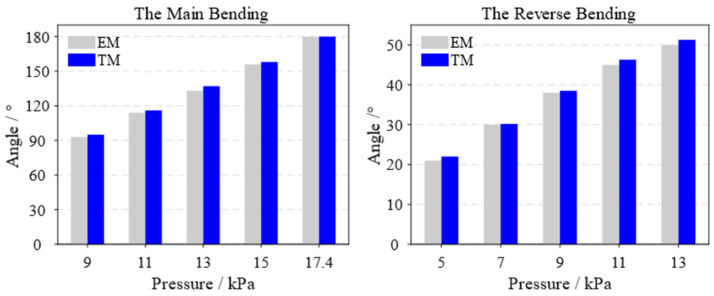
Bending angle values of the entity model and theoretical model of the soft actuator of the middle finger.

Using the middle finger as an example, the bending angles of both the EM and theoretical model (TM) of the soft actuator are shown in Figure 13, considering both the main and reverse bending under different air pressures. In the main bending condition, the deviation between the theoretical and entity models for the bending angle is less than 2.25%, based on the bending angle of the entity model. For the reverse bending, the value of the entity model is lower than the value of the theoretical model, with an error of less than 5%.

#### 4.1.2. Tip Force Measurement

**Figure 14:**
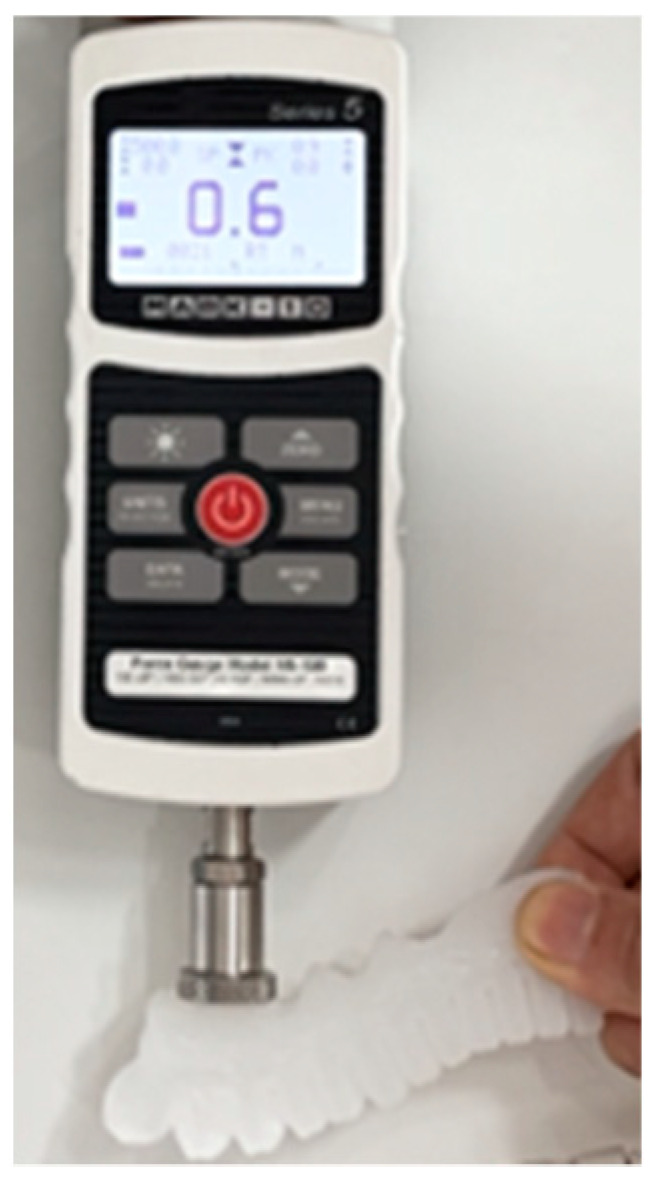
Fingertip force measuring device.

Functional grasping requires sufficient fingertip force and proper directional control, which are crucial for activating the relevant hand muscles and are essential for rehabilitation [47]. Therefore, the fingertip force was measured for different soft actuators via a force gauge (M5-100, MARK-10 Series 5, Copiague, NY, USA), as shown in Figure 14.

**Figure 15:**
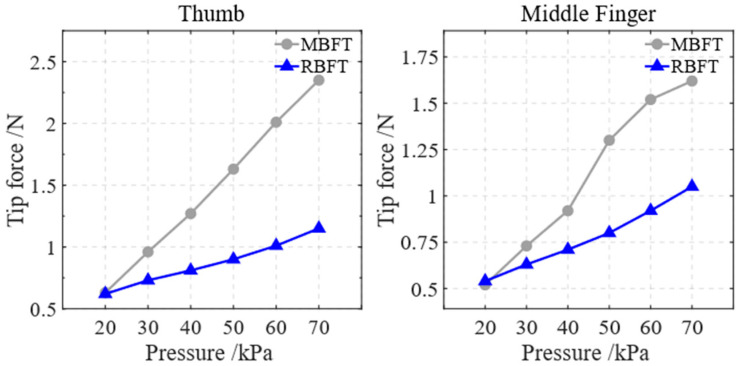
Fingertip forces for main and reverse bending of the soft actuator at different air pressures.

The air pressure within the soft actuator was varied from 20 kPa to 70 kPa in 10 kPa increments. The fingertip force for both main bending (MBFT) and reverse bending (RBFT) of the thumb and middle finger was measured at each air pressure level. The results are shown in Figure 15. Compared with the initial air pressure of 20 kPa, when the starting air pressure was increased to 70 kPa, the fingertip force for the main bending of the thumb increased to 2.35 N, a 273% increase. The fingertip force for reverse bending of the thumb reached 1.15 N, representing an 85.5% increase. The fingertip force for the main bending of the middle finger reached 1.62 N, a 211.5% increase. The fingertip force for reverse bending of the middle finger was 1.05 N, a 94% increase. The experimental results indicate that as air pressure is applied, the fingertip force increases, demonstrating the load-bearing capacity of the soft actuator.

**Figure 16:**
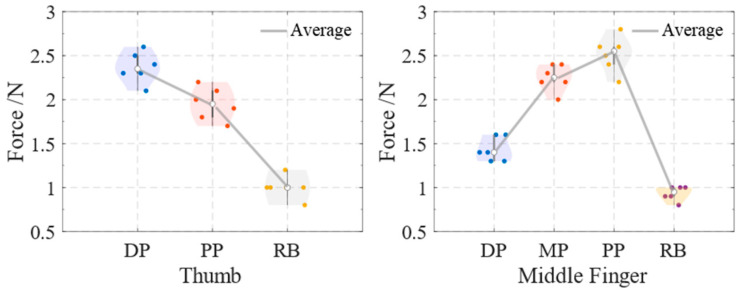
Forces at each joint and reverse-bending fingertip forces of the soft actuators under air pressure of 70 kPa.

Additionally, at an air pressure of 70 kPa, the maximum force of the joints (DP, PP) of the thumb and the maximum fingertip force of the overall reverse bending of the thumb in the soft actuator were measured. The maximum force of the joints (DP, MP and PP) of the middle finger and the maximum fingertip force of the overall reverse bending of the middle finger in the soft actuator were also measured. As shown in Figure 16, six experiments were conducted. The maximum force at each joint of the soft actuator was consistently greater than the maximum fingertip force generated during reverse bending. The average forces were as follows: for the thumb, 2.37 N at the DP, 1.95 N at the PP, and 1 N for the reverse-bending fingertip force; for the middle finger, 1.4 N at the DP, 2.25 N at the MP, 2.55 N at the PP, and 0.95 N for the reverse-bending fingertip force. The reverse fingertip force of the soft actuator designed by F. Wang et al. is 0.6 N under an air pressure of 70 kPa, and the main fingertip force of the soft actuator is 1.59 N under an air pressure of 130 kPa [26].

### 4.2. Soft Glove Testing

**Figure 17:**
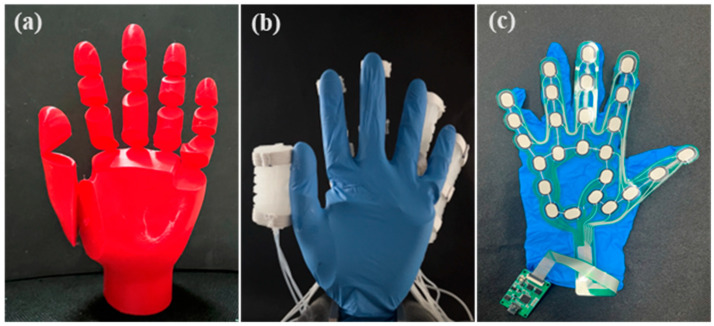
The prosthetic model and initial state of wearing the soft glove. (**a**) the prosthetic model; (**b**) the initial state of the prosthetic model wearing the soft glove; (**c**) the thin-film pressure sensor.

The soft glove was constructed by attaching the soft actuators to a nitrile glove via nylon cords. To evaluate the performance of the pneumatic soft glove, we created a prosthetic hand model, as shown in Figure 17a. On the basis of the proportions and joint range of motion (ROM) of a human hand, a joint opening of 60° and a connection thickness of 1 mm were designed. The model was then 3D-printed using thermoplastic urethane (TPU) material. The soft robotic glove was worn on the prosthetic hand, with a thin-film pressure sensor (RX-G0505M, Roxifsr, Changzhou, China) placed inside the glove, as shown in Figure 17b. The thin-film pressure sensor is illustrated in Figure 17c.

#### 4.2.1. Range-of-Motion Test for the Fingers in the Soft Glove

To evaluate the impact of the soft glove on the finger range of motion, the glove was worn on both the prosthetic hand model and a human hand for comparison. The flexion angles of the index, middle, and ring fingers on both the prosthetic hand and the human hand were measured at different air pressures.

**Figure 18:**
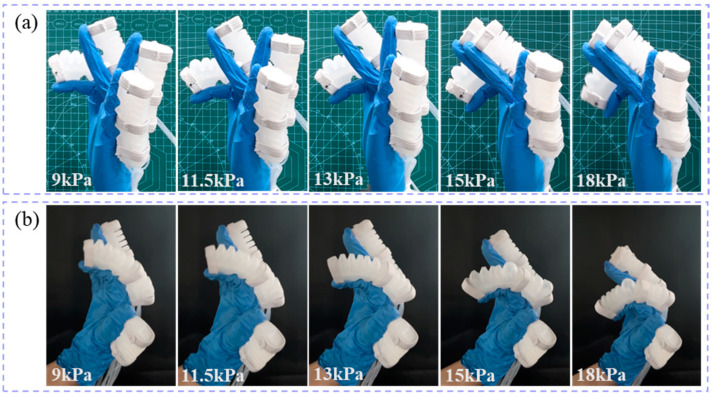
The flexion states of the index finger under different air pressures. (**a**) the prosthetic hand model; (**b**) the human hand.

**Figure 19:**
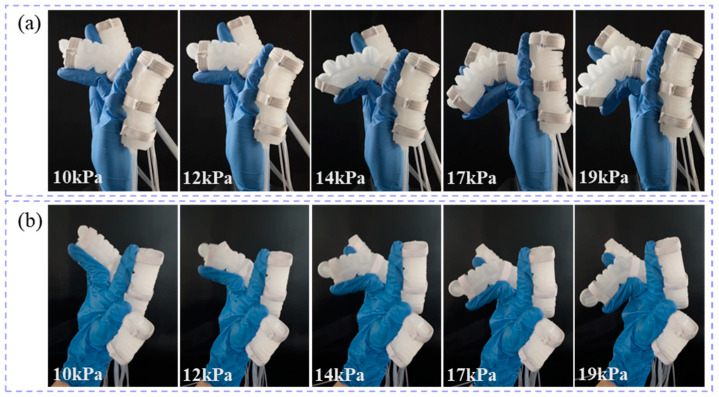
The flexion states of the middle finger under different air pressures. (**a**) the prosthetic hand model; (**b**) the human hand.

**Figure 20:**
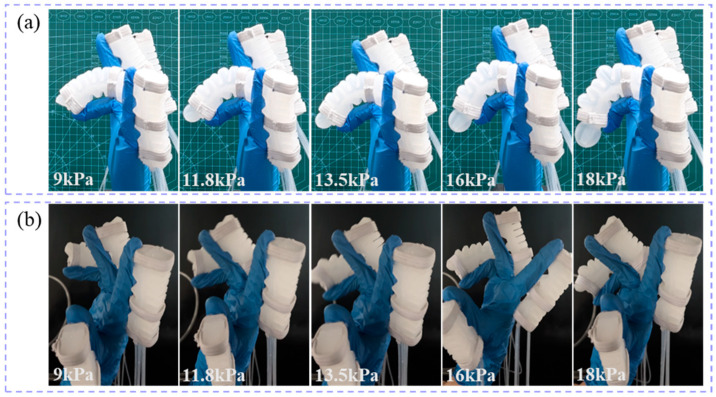
The flexion states of the ring finger under different air pressures. (**a**) the prosthetic hand model; (**b**) the human hand.

The flexion states of the index finger on the prosthetic hand model (Figure 18a) and the human hand (Figure 18b) at air pressures of 9 kPa, 11.5 kPa, 13 kPa, 15 kPa, and 18 kPa are shown in Figure 18. Similarly, the flexion states of the middle finger on the prosthetic hand model (Figure 19a) and the human hand (Figure 19b) at air pressures of 9 kPa, 11.5 kPa, 13 kPa, 15 kPa, and 18 kPa are shown in Figure 19. Finally, the flexion states of the ring finger on the prosthetic hand model (Figure 20a) and the human hand (Figure 20b) at air pressures of 9 kPa, 11.5 kPa, 13 kPa, 15 kPa, and 18 kPa are shown in Figure 20.

**Figure 21:**
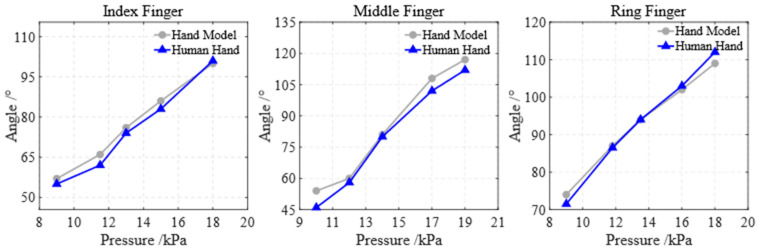
Finger flexion angles of both the prosthetic hand model and the human hand.

The finger flexion angles (the angle between the base and tip of the finger) for both the prosthetic hand model and the human hand are plotted in Figure 21. The experimental results demonstrate that the soft glove allows the index, middle, and ring fingers to achieve flexion angles of 101°, 112°, and 112°, respectively, indicating its strong practicality and biomimetic performance.

#### 4.2.2. Rehabilitation Gesture Testing of the Soft Glove

**Figure 22:**
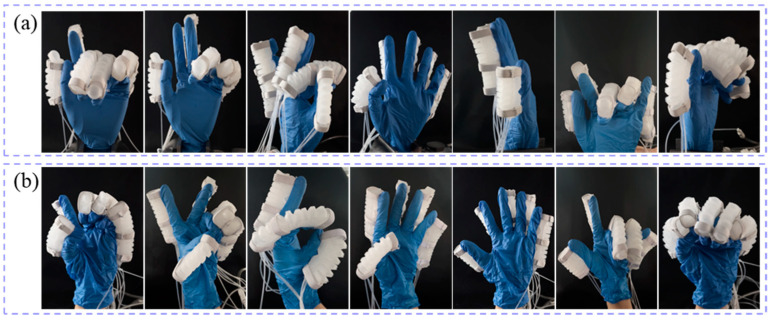
Soft glove-assisted rehabilitation gestures. (**a**) the prosthetic hand model; (**b**) human hand.

Under the control of the pneumatic platform, both the prosthetic hand model and the human hand, worn with the soft glove, are capable of performing various rehabilitation gestures, as shown in Figure 22. The initial state of the prosthetic hand model wearing the soft glove is shown in Figure 17b. With the assistance of the soft glove, both the prosthetic hand model (Figure 22a) and the human hand (Figure 22b) successfully performed various gestures, including “1”, “2”, “3”, “4”, “5”, “love”, and “fist.” The experimental results demonstrate that the pneumatic soft glove has the potential to support patients with hand impairments in training for a variety of rehabilitation gestures.

#### 4.2.3. Test of the Auxiliary Grip

**Figure 23:**
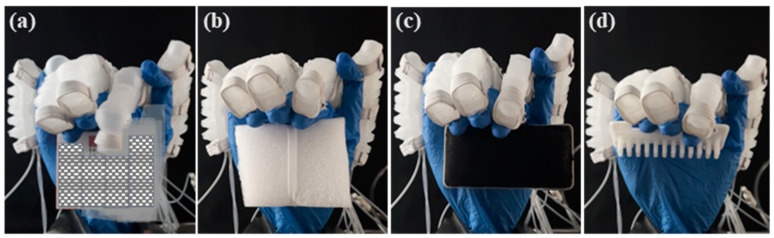
Gripping objects by prosthetic hand with soft glove. (**a**) a rectangular box; (**b**) a foam; (**c**) a blackboard eraser; (**d**) a PLA mold.

The soft glove assists the prosthetic hand in grasping objects of varying weights and shapes, as shown in Figure 23. The objects grasped included a rectangular box (15.4 g), foam (3.8 g), a blackboard eraser (18.5 g), and a PLA mold (19.2 g). The experimental results demonstrate that the pneumatic soft glove can assist the hand to grasp objects.

**Figure 24:**
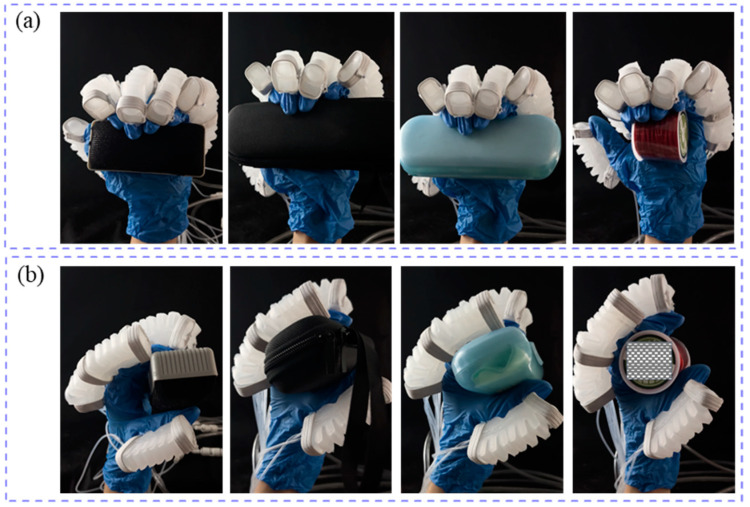
Grasping objects with two grasping postures by human hand wearing soft glove. (**a**) full grip; (**b**) flat pinch.

By using a pneumatic soft glove to assist with daily tasks, meaningful grasping exercises can be designed to enhance hand function in stroke patients [48]. Two different grasping techniques, full grip (FG) and flat pinch (FP) were employed to handle objects of varying shapes and weights. The pneumatic soft glove assisted the human hand in grasping tasks involving a blackboard eraser (18.5 g), U-disk box (139.4 g), glasses case (38.4 g), and coil (22.6 g), as shown in Figure 24. Figure 24a shows the FG scenes, whereas Figure 24b shows the FP scenes. The experimental results indicate that the soft robotic glove can grasp objects weighing at least 139.4 g.

**Figure 25:**
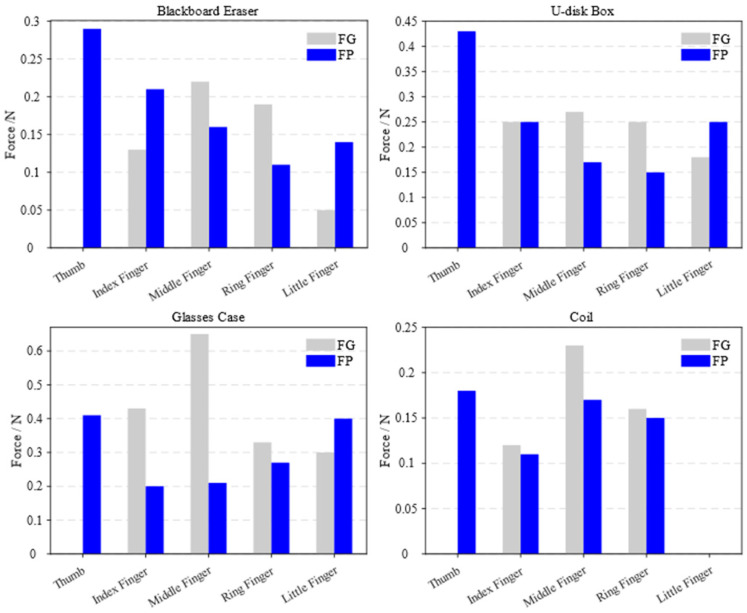
Five-finger tip forces through two grasp types.

The fingertip forces of all five fingers were measured by a thin-film pressure sensor during the grasping of different objects with the two grip techniques, as shown in Figure 25. The experimental results indicate that the pneumatic soft glove can assist patients with hand impairments in performing various daily tasks.

## 5. Conclusions

This work presents a novel pneumatic soft glove with bidirectional bending functionality. It can perform various rehabilitation gestures and grasping tasks. An expandable pneumatic control platform is developed, enabling the soft glove to assist patients with hand impairments in rehabilitation exercises and daily activities. Experiments show that the soft actuator possesses a measurable load capacity and that the effect of gravity on the bending angle and air pressure of the soft actuator is negligible, enabling not only finger flexion and extension but also grasping functionality. The maximum main and reverse-bending angles of the soft actuator are sufficient to meet the requirements of daily human activities. In addition, a prosthetic hand model was designed and a soft glove was tested on both the prosthetic hand and the human hand, verifying that the soft glove system is capable of enabling individual finger movement, performing various rehabilitation gestures, and assisting with grasping tasks. The experimental results indicate that the pneumatic soft glove has the potential to support patients with hand impairments in various rehabilitation exercises and enable them to grasp different objects to complete daily tasks.

In future work, we will focus on designing a versatile pneumatic soft glove with bidirectional bending capabilities and exploring new materials or structures to increase the grasping force, thereby enabling the completion of a broader range of daily tasks. Furthermore, computer vision will be incorporated into the motion data collection of the soft glove to obtain more precise and comprehensive experimental results. Finally, electromyographic (EMG) signals will be utilized for detecting user intent and controlling the movements of the soft glove, improving the accuracy of the movements of glove and providing better support for stroke patients with hand impairments.

## Figures and Tables

**Table 1:** The material parameters of Ecoflex 00-50.

Material Characteristics	Ecoflex 00-50
Specific Gravity	1.07
Cure Time	3 h
Shore Hardness	00-50
Tensile Strength	2.172 MPa
100% Modulus	0.083 MPa
Elongation at Break %	980%

## Data Availability

The data that support the findings of this study are available from the authors upon reasonable request.

## Appendix A

**Table A1:** Parameters of the soft actuator.

Values	Parameters
a=3.6 mm	Length of a single air chamber of the main bending module
h=9.75 mm	Height of the air chamber of the main bending module
c=10 mm	Effective width of the soft actuator
$a_{1}=1.88$ mm	Length of the short side of the isosceles trapezoid of the reverse-bending module
$b_{1}=10.8$ mm	Length of the long side of the isosceles trapezoid of the reverse-bending module
$h_{1}=5.24$ mm	Height of the air cavity in the reverse-bending module
α=49.5°	Bottom corner of a trapezoid
T=23 °C	Normal temperature
E=0.1 MPa	Young’s modulus of Ecoflex 00-50
G=0.044 MPa	Shear modulus of Ecoflex 00-50

**Table A2:** Model and origin of components for pneumatic control platform.

Components and Parts	Model	Brand	Place of Origin
Pressure-relief valve	IR2020	SMC	Tokyo, Japan
Buck	LM2596S	-	China
Controller	A Tmega2560	KEYES	Shenzhen, China
2/2-way solenoid valve	2V025	AirTac	Taiwan, China
Air pressure sensor	MSP20	-	China
2/3-way solenoid valve	SF3	PIN YA	Shenzhen, China
2/3-way solenoid valve	VT307	SMC	Tokyo, Japan
